# Anti-*Blastocystis* Activity *In Vitro* of Egyptian Herbal Extracts (Family: Asteraceae) with Emphasis on *Artemisia judaica*

**DOI:** 10.3390/ijerph16091555

**Published:** 2019-05-03

**Authors:** Amira B. Mokhtar, Shahira A. Ahmed, Enas E. Eltamany, Panagiotis Karanis

**Affiliations:** 1 Department of Medical Parasitology, Faculty of Medicine, Suez Canal University, Ismailia 45122, Egypt; shahira_ahmed@med.suez.edu.eg; 2 Department of Clinical Laboratory Sciences, College of Applied Medical Sciences, Jouf University, Al-Qurayyat 77413, Saudi Arabia; 3 Department of Pharmacognosy, Faculty of Pharmacy, Suez Canal University, Ismailia 45122, Egypt; enastamany@gmail.com; 4 University of Cologne, Medical Faculty and University Hospital, 50937 Cologne, Germany; panagiotis.karanis@uk-koeln.de

**Keywords:** *Blastocystis*, Asteraceae, *Artemisia judaica*, fractionation

## Abstract

*Achillea fragrantissima* (Forssk.) Sch. Bip. (known as Qaysoom), *Echinops spinosus* L. (known as Shoak Elgamal) and *Artemisia judaica* L. (known Shih Baladi) are members of the Asteraceae family known for their traditional medical use in Egypt. The ethanol extracts of these plants were evaluated for their efficacy against a protozoan parasite (*Blastocystis*). Two different molecular subtypes of *Blastocystis* were used (ST1 and ST3). Significant growth inhibition of *Blastocystis* was observed when exposed to both *A. judaica* (99.3%) and *A. fragrantissima* (95.6%) with minimal inhibitory concentration (MIC_90_) at 2000 µg/mL. Under the effect of the extracts, changes in *Blastocystis* morphology were noted, with the complete destruction of *Blastocystis* forms after 72 h with the dose of 4000 µg/mL. Different subtypes displayed different responses to the herbal extracts tested. ST1 exhibited significantly different responses to the herbal extracts compared to ST3. *A. judaica* was selected as the herb of choice considering all of its variables and because of its effective action against *Blastocystis*. It was then exposed to further fractionation and observation of its effect on ST1 and ST3. Solvent portioned fractions (dichloromethane (DCM), ethyl acetate (EtOAc) and n-hexane) in *A. judaica* were found to be the potent active fractions against both of the *Blastocystis* subtypes used.

## 1. Introduction

*Blastocystis* species, anaerobic intestinal parasites, are one of the commonly detected parasites in human stool samples as well as in a variety of non-human hosts [1,2]. It is widely distributed throughout the world, with a high frequency in developing countries that is linked to the consumption of contaminated food or water, poor hygienic practices and exposure to animals [3,4]. *Blastocystis* prevalence can reach up to 30% in industrialized countries, whereas in developing countries it extends to 100% [5,6].

The pathogenic role of *Blastocystis* causing disease in humans still remains controversial. Some scientists argue about the harmfulness of the parasite in humans due to its association with asymptomatic carriers [7], while others support the opinion of the potential pathogenic role it may play in symptomatic cases [8]. Seventeen distinct subtypes (STs) of the parasite have been identified using the molecular analysis of the small subunit rRNA (SSU-rRNA) gene. Although ST1–ST9 have been detected in humans, ST1–ST4 are the most prevalent and they represent about 90% of the infections in humans [9,10]. *Blastocystis* has been implicated as a possible cause of irritable bowel syndrome, inflammatory bowel disease, chronic diarrhoea and ulcerative colitis [11,12,13]. Other extra intestinal diseases, such as urticaria, chronic angioedema, anaemia, chronic liver disease and reactive arthritis have also been reported to be potentially associated with *Blastocystis* infection [14,15,16,17,18]. *Blastocystis* has also been reported to have an opportunistic role in immunocompromised individuals (cancer, organ transplant and human immunodeficiency virus patients) [19,20].

Recently, *Blastocystis’* resistance to the most commonly used drugs has become a topic of discussion for researchers [21,22]. Herbal medicine has many potential benefits as it may be safer, healthier and cheaper. The Asteraceae family is the largest and the most widely distributed family and its plants are especially abundant in tropical and subtropical regions [23,24]. In Egypt, this family has about 14 genera and 230 species [25]. Members of the Asteraceae family have been reported to have various therapeutic activities, including anti-pyretic, anti-inflammatory, anti-oxidising, detoxifying, anti-tumor, anti-bacterial, wound-healing, anti-hemorrhagic, antalgic, anti-tussive and anti-spasmodic [26]. The Asteraceae family has a great reputation in the production of sesquiterpenoid and sesquiterpene lactones, which possess anti-parasitic, anti-tumour, anti-bacterial, anti-fungal, cytotoxic and insecticidal properties [27,28].

Artemisinin is an endoperoxide sesquiterpene lactone compound. It is an important constitute of *Artemisia annua* that retained potential anti-malarial activity [24,29]. Terpenoidal compounds and volatile oils are well known for their anti-microbial potential [30]. Flavonoids have also been reported to have an extensive anti-oxidant and protective effect against cancer, and some of them are promising anti-protozoal agents [31]. *Achillea fragrantissima* (Forssk.) Sch. Bip. (known as Qaysoom), *Echinops spinosus* L. (*Echinops spinosissimus* Turra) (known as Shoak Elgamal) and *Artemisia judaica* L. (known as Shih Baladi) are Asteraceae plants grown in the Egyptian deserts. *A. fragrantissima* is a fragrant perennial herb used by Bedouins as an anti-helminthic, anti-diabetic [32], anti-ulcerogenic [33] and anti-spasmodic [34]. *E. spinosus* is a perennial spiny herb; its preparations were used in traditional medicine as an abortifacient, anti-diabetic and diuretic and to improve blood circulation [35]. *A. judaica* is a perennial aromatic shrub commonly used as an anti-helminthic and reported to have anti-microbial, anti-inflammatory, anti-oxidant [36] and anti-diabetic effects [37]. No previous studies have investigated the anti-protozoal potential of these herbs on *Blastocystis* spp. yet.

The aim of this study was to examine the *in vitro* susceptibility of different clinical isolates from *Blastocystis,* along with the ethanolic extract of three plants from the Asteraceae family (*A. fragrantissima, E. spinosus* and *A. judaica*) in comparison to metronidazole (as a reference drug). Thereafter, the herbal extract that has the highest efficacy on *Blastocystis* spp. would then be exposed to the solvent partitioned fractionation.

## 2. Materials and Methods

### 2.1. Source of Faecal Samples

Eleven faecal specimens were obtained from patients with gastrointestinal symptoms. The samples were provided in a fresh state by the Ismailia lab for medical tests. Directly upon arrival, a small part of each specimen (a pea-sized amount of the formed stool/0.5 mL of a diarrhoea stool) was cultured in previously prepared Jones’ medium. The remaining part of the faecal samples was then processed with formalin-ethyl acetate concentration. The concentrate was examined with wet mount, an iodine stain, a trichrome stain and a modified trichrome stain to exclude the possibility of a mixed infection with other parasites. Microbiological examination of samples was then performed. The faecal samples were cultured on Xylose Lysine Deoxycholate and MacConkey media to exclude gram negative bacteria (*Salmonella* spp. and *Shigella* spp.). Only the faecal samples that contained the *Blastocystis* spp. infection (two samples) were selected for further analysis (Table 1).

### 2.2. Cultivation of Blastocystis spp. In Vitro

The faecal specimens were cultured in Jones’ medium without rice starch and containing 10% horse serum, 100 UI/mL penicillin and 100 μg/mL streptomycin at 37 °C [38]. No starch was added to the Jones’ medium. The *Blastocystis* multiplication rate was screened with light microscopy every 2–3 days. When the typical vacuolar/granular forms of *Blastocystis* were observed, they were sub-cultured in a new medium. Repeated subculture in a new medium was maintained for one month in order to cleanse the culture medium from faecal debris. About 0.5 mL of the *Blastocystis* sediment was then transferred to Eppendorf tubes and frozen at −20 °C until DNA extraction.

### 2.3. Molecular Identification of Blastocystis spp. Subtypes

To extract DNA, about 200 µL of the frozen culture concentrate was thawed with InhibitEX buffer. The mixture was then further processed according to the protocol of the QIAamp Fast DNA Stool Mini Kit (Qiagen, Hilden, Germany). Isolated DNA was kept at −20 °C until PCR procedure.

Conventional PCR was used to genetically identify the subtypes of *Blastocystis* spp. in the two samples that were previously selected. Seven primer pairs were used to amplify the SSU rRNA gene of *Blastocystis* (Table 2). The PCR amplification system was performed in a total volume of 25 µL, containing 12.5 µL master mix (Applied Biotechnology Co. Ltd, Cairo, Egypt), 20 pM of each primer pair (each subtype primer in a separate PCR tube), 9.5 µL nuclease-free water and 1 µL of template DNA. The conditions of the PCR were adapted according to Yoshikawa et al. [39] (Table 2). In brief, the PCR amplification was performed with 35 cycles of initial denaturation at 94 °C for 3 min, followed by 30 cycles of denaturation at 94 °C for 30 seconds, annealing at 57 °C for 30 seconds and then extension at 72 °C for 1 min, followed by an additional cycle with a 10-min chain elongation at 72 °C. The PCR product was electrophoresed at 1.5% agarose stained with ethidium bromide using a size marker of 100 bp ladder (Applied Biotechnology Co. Ltd, Cairo, Egypt). The electrophoresed products were then visualized using a UV transilluminator [39].

### 2.4. Collection of Plant Materials

*A. fragrantissima* aerial parts were collected in June from El Arbaen valley, South of Sinai Peninsula. *E. spinosus* aerial parts were collected from the International Coastal Road near Kafr Elsheikh Governorate in the early summer. *A. judaica* aerial parts were purchased from Egyptian markets. The plants were authenticated by Abdelraof A. Mostafa and Elsayeda M. Gamal El-Din, Department of Botany, Faculty of Science, Suez Canal University. The voucher specimens of *A. fragrantissima*, *E. spinosus* and *A. judaica* were then deposited in the Pharmacognosy Department Herbarium, Faculty of Pharmacy, Suez Canal University, Ismailia, Egypt, under registration numbers of Af-2006-1, Es-2016-1 and Aj-2018-1, respectively.

### 2.5. Plants Extraction

The collected plants were dried at room temperature and then powdered; 200 g of each herb was then extracted three times with 300 mL of 95% ethanol for 48 h. The extracts were concentrated under reduced pressure at 40 °C to yield 14 g of *A. fragrantissima*, 26 g of *E. spinosus* and 17 g of *A. judaica* crude extracts.

### 2.6. Use of Metronidazole as a Reference Anti-Protozoal Drug

In these experiments, metronidazole (MTZ) was used as a reference anti-protozoal drug for further comparison with the results of the herbal extracts used. The stock solution was used to prepare different concentrations of MTZ (5, 10, 20 µg/mL) [40].

### 2.7. In Vitro Exposure of Blastocystis spp. to Egyptian Herbal Extracts

All experimental steps were performed in 1.5-mL Eppendorf tubes using a cell suspension of 2 × 10^5^/mL *Blastocystis*. The *Blastocystis* cell suspension was prepared from protozoal Jones’ cultures that were grown for up to 48 h prior to testing. The total number of live parasites was counted in a Neubauer cell counting chamber by vital coloration using 0.4% Trypan blue solution. In each test tube, the *Blastocystis* suspension and the corresponding concentration of the herbal extracts or the reference drug was calculated to a final volume of 1 mL.

The three different Egyptian herbal extracts (*A. fragrantissima, E. spinosus, A. judaica*) were dissolved in 70% ethanol and trilled against the *Blastocystis* parasite at different concentrations (250, 500, 1000, 2000, 4000 µg/mL). Each herbal extract concentration was tested in twice. The minimal inhibitory concentration (MIC_90_), defined as the lowest concentration of herbal extract in which 90% of *Blastocystis* growth was inhibited. MIC_90_ was calculated for each extract separately.

A control tube was included, and it consisted of 2 × 10^5^ *Blastocystis* suspension/mL in a cell culture medium without the addition of the herbal extract. The tubes were then incubated at 37 °C for 72 h. The use of 70% ethanol as a solvent for the herbal extracts was proven to have no significant effect on *Blastocystis* parasite growth [40].

### 2.8. Counting the Treated Blastocystis in Suspensions to Examine the Efficacy of the Herbal Extracts Tested

The efficacy of the herbal extracts against *Blastocystis* was observed at 24, 48, and 72 h. A Neubauer haemocytometer chamber was used to count the number of living *Blastocystis* cells, revealed by Trypan blue under a light microscope at ×40. Non-viable cells were stained blue, whereas viable ones remained unstained. An oil lens was used to confirm the uncertain non-viability status of some of the cells at ×40 according to presence of intact cell wall and destructed/non-destructed internal content. The mean of two counted chambers was compared against the control.

The percentage of the parasite reduction was calculated according to the growth inhibition equation (A − B/A) × 100, where A = mean number of intact viable cells in control tube, while B = mean number of intact viable cells in treated tubes. Complete inhibition (100%) of growth was confirmed on day 7 by the inoculation of the respective *Blastocystis* suspension into a fresh culture medium without the addition of any herbal extract compounds.

#### 2.9. Fractionation of A. judaica Extract

Dried *A. judaica* extract was defatted by partitioning between n-hexane and water. The aqueous layer was then concentrated and partitioned successively with dichloromethane (DCM), ethyl acetate (EtOAc) and n-butanol (n-BuOH). Water-soluble fraction and all solvent extracts were then concentrated under vacuum. The purified fractions were observed for their immediate activity (within 30 minutes) against the two *Blastocystis* isolates using 100 µg/mL culture media for each fraction.

### 2.10. Statistics

The statistical significance of the variations between the extract susceptibility values of the two isolates at different time periods was determined using a two-way analysis of variance (ANOVA), and the fractionation values were determined using a one-way ANOVA. The ANOVA test is ideal to test the statistical significance of the variations observed between means of data. In this study, *p* values of ≤0.05 were considered significant. All the analyses were done with the IBM SPSS Statistics V23.0 (IBM Corp., Armonk, NY, USA).

## 3. Results

### 3.1. Blastocystis spp. Isolates and Its Molecular Identification

The two isolates in this study were obtained from symptomatic patients suffering from gastrointestinal symptoms who were not undergoing any treatment. Under light microscopy the faecal samples were examined and were found to be infected with vacuolar forms of *Blastocystis* (Table 1). The infection was confirmed with a trichrome stain. There was no mixed infection with any other parasitic species. The microbiological examination of the two faecal samples for gram negative bacterial infection was negative. The two isolates were also successfully cultured and maintained in Jones’ medium.

The two Egyptian isolates were genetically identified using seven sets of primers. They were amplified with only one of the distinct STs of primers. Based on the PCR amplification, the observed genotypes were ST1 and ST3 (Table 1 and Table 2).

### 3.2. Anti-Blastocystis Activity

The three herbal plants (*A. fragrantissima, E. spinosus* and *A. judaica*) revealed anti-*Blastocystis* activity from their ethanolic extracts. The MIC_90_ values of the three substances are displayed in Table 3, presenting their anti-*Blastocystis* activities compared with the MIC_90_ of MTZ (the anti-protozoal reference drug). Dose-response effect was observed when the doses of the extracts were doubled. The MIC_90_ was observed at a concentration of 2000 µg/mL. At the concentration of 2000 µg/mL, *A. judaica* displayed the highest activity against *Blastocystis*, with 99.3% inhibition of growth, followed by *A. fragrantissima* with 95.6%, whereas *E. spinosus* did not display significant activity against *Blastocystis*.

The inhibition of *Blastocystis* growth reached its peak with all of the herbal extracts at the dose of 4000 µg/mL, where the complete death of *Blastocystis* cultured stages was observed and remained for 7 consecutive days.

The *Blastocystis* growth reacted differently during various times of the assessment (24, 48, 72 h). *Blastocystis* cells reacted well to the herbal extracts after 24 h and 48 h, however its reaction was reduced at 72 h. This phenomenon appeared with all of the extracts and with MTZ. MTZ displayed better inhibitory activity at lower concentrations against *Blastocystis* than any of the other tested plant extracts.

The Trypan blue stain facilitated the identification of viable and non-viable *Blastocystis* cells on the lowest magnification (×10). After extract exposure, *Blastocystis* cells were easily observed undergoing morphological changes.

### 3.3. Subtype-Dependent Variations of Blastocystis

**Figure 1:**
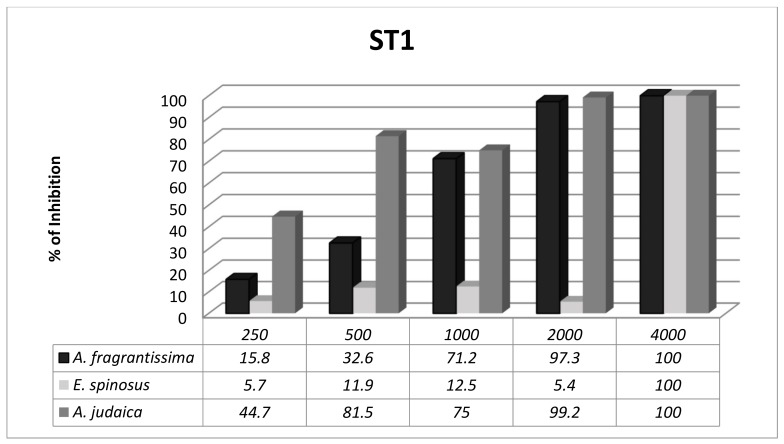
Active response of *Blastocystis* ST1 (percentage of growth inhibition) to different concentrations of the three herbal extracts at 72 h.

**Figure 2:**
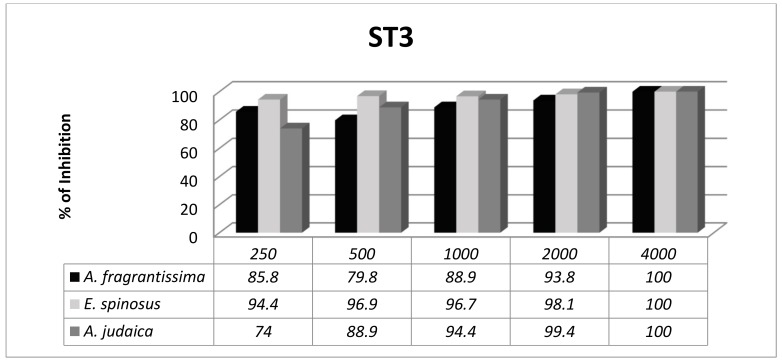
Active response of *Blastocystis* ST3 (percentage of growth inhibition) to different concentrations of the three herbal extracts at 72 h.

*Blastocystis* exhibited subtype-dependent variations in susceptibility to the herbal extracts. The anti-*Blastocystis* activity of the Asteraceae family was evaluated in vitro against two different subtypes of *Blastocystis* (ST1 and ST3). The significance of the results was evaluated at 72 h. There was an overall significant difference between the three herbal extracts at different concentrations with ST1 (Figure 1); however, ST3 was more sensitive to the herbal extracts, with no significant difference between the herbal extracts at different concentrations (Figure 2).

*A. judaica* was the most active extract against both subtypes (ST1 and ST3) according to MIC_90_. It was therefore selected for further identification of its active fractions.

### 3.4. Identification of Active Fractions in A. judaica

The multiple fractions of *A. judaica* were examined for their immediate cytotoxic potential against *Blastocystis.* Amongst the five fractions tested, the EtOAc, DCM and n-hexane exhibited the highest cytotoxic effect on both *Blastocystis* isolates (ST1 and ST3). For these fractions, 100 µg/mL caused complete death of *Blastocystis* in culture within 30 min, with significant statistical differences compared with n-BuOH and water-soluble fraction (Table 4).

## 4. Discussion

In developing countries, *Blastocystis* is not on the list of routine diagnoses and there is also no consensus regarding its treatment [3]. In Egypt, very few labs are capable of diagnosing *Blastocystis* in faecal samples. Even though many parasitologists have insisted that treatment of *Blastocystis* should be proposed when it is present in large numbers in stool examinations [41,42], many Egyptian medical doctors recommend no treatment at all.

Increasing incidence of *Blastocystis* resistance has become a problem, particularly to the most common anti-protozoal drugs, including MTZ. Moreover, this drug is able to cause undesirable changes in the gut microbiota with various potential side effects, including embryotoxic, carcinogenic and teratogenic [7]. Different susceptibilities of different *Blastocystis* subtypes, over-prescription and misuse of anti-microbial drugs might be some reasons for the treatment failure [21]. Investigating new anti-*Blastocystis* substances in research could overcome its resistance frequencies, particularly for substances of natural origin. Herbal plants are a cost-effective source of biological active metabolites that have therapeutic potential in the treatment of different diseases [43].

In the present study, the tested herbal extracts, *A. fragrantissima, E. spinosus* and *A. judaica*, were examples of natural home remedies. These herbs are typically consumed in Egypt as a decoction or infusion and sometimes mixed with other flavours to treat gastrointestinal disorders. We used the extracts of the three herbs to evaluate their anti-*Blastocystis* activities. The ethanolic extract was selected in particular based on its previous record of being the most effective preparation among other extracts, such as water extracts, heptane extracts and raw materials. This was clarified with observations of its better solubility in culture medium [44].

The three herbal extracts did not display identical activity against *Blastocystis*. *A. judaica* and *A. fragrantissima* were found to be the most effective extracts against *Blastocystis*. *A. fragrantissima* aerial parts required collection from wild desert places and were not available in Egyptian markets. Bedouins living in the deserts of Sinai, the location of *A. fragrantissima*, are the people with the greatest access to this herb. *A. judaica* was therefore selected as the extract of choice considering all of the variables within the herb itself (accessibility and price) and because of its action against *Blastocystis* (mean of count, concentration and diversity of *Blastocystis* STs). *A. judaica* is a cost-effective edible herbal plant with a high availability in Egyptian markets, thus explaining its wide presence in almost every Egyptian house. Under the Arabic name of Shih Baladi, *A. judaica* is used to treat helminth infections in the most of North African and Middle East countries [45] because of its anti-helminthic activity. It has been used as an insecticidal, anti-diabetic and anti-bacterial substance [37,45,46], however, the efficacy of the species has not been tested as of yet against protozoans.

The active concentration of *A. judaica* that inhibited 99.3% of *Blastocystis* growth was 2000 µg/mL (Table 3). Similar results were documented in various studies using different herbal extracts against *Blastocystis* [47,48]. The effective concentration usually supports the safety of the herbal extract. *A. judaica* has been proven to be a non-toxic extract. The oral administration of the ethanolic extract of *A. judaica* failed to kill mice (with less than 7.5 g/kg body weight) within 24 h after oral administration [37].

Genus *Artemisia* belongs to the Asteraceae family, the largest family of the flowering plants, containing about 500 species [24], according to the Royal Botanic Gardens K and MBG (http://www.theplantlist.org/tpl/search?q=artemisia). Plants in the *Artemisia* genus are recognized for their medicinal value as being anti-protozoal. For example, *A. argyi* has been used as an anti-amoebic with amoebicidal and amoebistatic effects [49]. Other protozoans have also been treated with *Artemisia*, and its derivatives were isolated from *A. annua* with effective results against *Plasmodium*, *Toxoplasma*, *Cryptosporidium*, *Giardia* and *Trypanosoma* [50]. The ethanolic extracts of *A. kulbadica, A. ciniformis* and *A. santolina* displayed promising leishmanicidal activity [51].

*A. judaica* inhibited *Blastocystis* growth and also caused morphological changes in the stages of cultivation. Microscopically, the frequently detected vacuolar forms appeared rounded with their cytoplasm pushed to one side by a large central vacuole. The changes in the morphology of the cultured forms of *Blastocystis* also resulted in them being irregular in shape and losing their lustre. Such changes are an indication of poor viability of *Blastocystis*, with a kind of gradual death process that might occur serially. After 24 h, visible vacuolar compression was prominently observed in the cell culture. After 48 h, vacuolar forms turned into granular forms, with granules replacing the central vacuole, and after 72 h, granular forms were compressed cells, with destructed cell membranes, and most of them no longer appeared in the microscopic field. Similar changes have previously been reported with other herbal extracts [52,53], as observed by scanning and transmission electron microscopic studies.

The Trypan blue stain provided a reliable method for a viable cell count to determine the herbal activity against *Blastocystis* isolates. With Trypan blue, morphological changes of *Blastocystis* were clearly visible. In addition, it was easy to differentiate the viable cells (unstained cells) from the non-viable (stained blue) ones under low microscopic power field (×10), however, it would be necessary to use higher power fields (×40 and ×100) in the case of detailed requirements.

The anti-*Blastocystis* activity of *A. judaica* extract might be relative to the bioactive compounds present in the aerial part of the plant. Such bioactive substances were reported to act synergistically or independently [54]. Lipophilic sesquiterpene lactones have particularly been proven to increase the fluidity of the protozoan membrane, resulting in the uncontrolled efflux of ions and metabolites leading to cell death [55]. Other various explanations have also been proposed that differed according to the protozoan tested [43,50].

ST1 and ST3 are the most frequently detected subtypes worldwide and in Egypt as well [3,56]. In the present study, the subtypes that were tested (ST1 and ST3) had various outcomes in terms of herbal extract treatment (Figure 1 and Figure 2). ST3 was more sensitive to all of the extracts tested, while ST1 displayed various responses to the extracts with significant statistical differences. These results were consistent with previous reports from other researchers [40,52,57].

Strain to strain variation has been observed in the susceptibility of *Blastocystis* to a panel of conventional and experimental anti-protozoal agents [58]. The subtype-dependent variation is known for its role in *Blastocystis* pathogenicity. ST1, ST4 and ST7 have been associated with pathological alterations in humans, whereas ST2 and ST3 were considered non-pathogenic. Furthermore, intra- and inter-subtype variations have been reported [59]. Such variations might explain the difference in response from both of the *Blastocystis* subtypes to the same extract in vitro.

In this study, MTZ displayed better inhibitory effects on *Blastocystis* isolates than the extracts tested. MTZ is considered the ideal drug for anti-protozoal activity, even though its efficacy has been shown to range from 0–100% [60]. Similar studies proved MTZ to be superior over herbal extracts against *Blastocystis* [40,47,61]. Nevertheless, neither of these studies used MTZ-resistant *Blastocystis* subtypes. It would therefore be beneficial to test the effect of herbal medicinal extracts against *Blastocystis* subtypes with previously documented MTZ-resistance. The effectiveness of MTZ can be explained by its single compound formula, whereas a herbal extract contains multiple compounds. Therefore the fractionation and sub-fractionation of herbal extracts is an important factor that might result in different outcomes.

In this study, *A. judaica* ethanol crude extract was subjected to further fractionation by solvent partitioning. Using solvents with different polarity would facilitate the analysis of the phytochemicals [62]. The fractions obtained were evaluated for their activities against the *Blastocystis* subtypes. Our results revealed that the three effective fractions were the non-polar n-hexane and intermediate polar DCM and EtOAc.

Several terpenoidal compounds, polymethoxylated flavonoids, coumarins, acetylenes and sterols have been isolated from *Artemisia* species [24,63]. *A. judaica* was previously analysed phytochemically; the herb recorded high flavonoid and phenolic contents [64] as well as sesquiterpene lactones [65].

Flavonoids have an obvious role (direct or indirect) in disease prevention. There are plenty of evidences that flavonoids have an effect on parasitic diseases (such as malaria), cardiovascular diseases and cancer [66].

Phenolic compounds are also known to provide protection against a wide range of diseases, such as coronary heart disease, strokes and certain types of cancers. The phytochemical analysis of a Brazilian herbal extract revealed that phenolic compounds displayed anti-leishmanial activity due to the presence of quercetin, a potent known leishmanicidal compound [67]. Terpenoids, in particular sesquiterpene lactones and flavonoids are the major phytochemicals in the aerial parts of *A. judaica* and abound in lipophilic extracts [65]. Consequently, they could be specifically attributed to the anti-*Blastocystis* potential of hexane, DCM and EtOAc fractions. Usually, the reported mechanism of action of sesquiterpene lactones involves the presence of an α-methylene γ-lactone ring and an α, β unsaturated cyclopentanone ring [29].

The extract is a mixture of several compounds, in which chemical interactions can merge synergistically, antagonistically or indifferently and therefore alter the effect that each one would have on its own [67]. Further assays concerning the biological activity of the single components of the plant would therefore be of interest, as such assays may reduce side effects and also help to establish an understanding of the bio-assays of individual pure compounds and to recognize inconsistencies in the extract preparations.

## 5. Conclusions

*A. judaica* has promising anti-*Blastocystis* potential. Its low cost, safety and availability are advantages which contribute to its effectiveness as a treatment for blastocystosis. Different subtypes of *Blastocystis* with variable pathogenicity are undeniable. The *A. judaica* extract remains the best extract among the other herbs used to overcome variable *Blastocystis* subtypes. Fractionation is essential for a deep look at the herbal phytochemical analysis. It is imperative to recognize DCM, EtOAc and n-hexane as the potent fractions in *A. judaica*. Further bio-assay guided isolation is therefore necessary to justify the pure individual compounds of these fractions in order to utilize *A. judaica* in a therapeutic form for subsequent animal and human trials.

## Figures and Tables

**Table 1:** Demographic data of the two isolates with microscopic and molecular identification.

Isolate No.	Age of Patient	Sex	Symptoms	Since How Long	Stool Consistency	Microscopic Examination	Molecular Subtyping
Isolate 1	6 years	Male	Diarrhoea	3.5 months	Semi-formed	1–2 *Blastocystis* forms/field	ST1
Isolate 2	8 years	Female	Abdominal pain	7 months	Formed	4–5 *Blastocystis* forms/field	ST3

**Table 2:** PCR genotyping data of *Blastocystis* isolates.

Subtypes	STs Primer Sets	Primers	Expected PCR Size	PCR Results of This Study’s Isolates
ST1	SB83	F 5′-GAAGGACTCTCTGACGATGA-3′ R 5′-GTCCAAATGAAAGGCAGC-3′	351 bp	ST1
ST2	SB340	F 5′-TGTTCTTGTGTCTTCTCAGCTC-3′ R 5′-TTCTTTCACACTCCCGTCAT-3′	704 bp	-
ST3	SB227	F 5′-TAGGATTTGGTGTTTGGAGA-3′ R 5′-TTAGAAGTGAAGGAGATGGAAG-3′	526 bp	ST3
ST4	SB337	F 5′-GTCTTTCCCTGTCTATTCTGCA-3′ R 5′-AATTCGGTCTGCTTCTTCTG-3′	487 bp	-
ST5	SB336	F 5′-GTGGGTAGAGGAAGGAAAACA-3′ R 5′-AGAACAAGTCGATGAAGTGAGAT-3′	317 bp	-
ST6	SB332	F 5′-GCATCCAGACTACTATCAACATT-3′ R 5′-CCATTTTCAGACAACCACTTA-3′	338 bp	-
ST7	SB155	F 5′-ATCAGCCTACAATCTCCTC-3′ R 5′-ATCGCCACTTCTCCAAT-3′	650 bp	-

STs: subtypes; bp: base pair, -: no amplification.

**Table 3:** Anti-*Blastocystis* activity according to MIC_90_ of the three Egyptian herbal extracts and MTZ.

Plant Species	MIC_90_ of *Blastocystis* in Culture †							Degree of *Blastocystis* elimination *
	Conc. (µg/mL)	*Blastocystis* ×10^4^ cells/mL after 24 h (% Inhibition)		*Blastocystis* ×10^4^ cells/mL after 48 h (% Inhibition)		*Blastocystis* ×10^4^ cells/mL after 72 h (% Inhibition)		
*A. fragrantissima*	250	51.1	(41.7)	15	(80.3)	44.5	(48.6)	++
	500	46.8	(46.7)	13.9	(81.8)	39.3	(54.7)	++
	1000	22.9	(73.9)	13.5	(82.3)	17.75	(79.4)	+
	2000	5.9	(93.3)	3.8	(95)	3.75	(95.6)	-
	4000	0	(100)	0	(100)	0	(100)	-
*E. spinosus*	250	30.9	(64.8)	19.3	(74.7)	45.6	(47.3)	++
	500	33.8	(61.5)	18.5	(75.8)	41.9	(54.7)	++
	1000	23.5	(73.2)	8.9	(88.2)	41.5	(52)	++
	2000	12	(86.3)	29.3	(61.6)	44.5	(48.6)	++
	4000	0	(100)	0	(100)	0	(100)	-
*A. judaica*	250	41	(53.3)	11.1	(85.5)	36	(58.4)	++
	500	23.4	(73.4)	7.25	(90.4)	13	(85)	+
	1000	23.9	(72.8)	6	(92.1)	13.9	(83.9)	+
	2000	2.9	(96.7)	0.4	(99.4)	0.5	(99.3)	-
	4000	0	(100)	0	(100)	0	(100)	-
MTZ (Reference drug)	5	14	(84)	12.5	(83.6)	9.5	(89)	+
	10	2.5	(97.2)	7.6	(90)	14.75	(82.8)	+
	20	0.5	(99.4)	0	(100)	0	(100)	-

† Numbers represent the mean counts (×10^4^) of *Blastocystis* in cultures treated with the extracts and the growth inhibition is the result of (A−B/A) × 100, where A = mean number of intact viable cells in control tubes, while B = mean number of intact viable cells in treated culture tubes.* Degree of *Blastocystis* elimination is based on the results after 72 h. -: No viable *Blastocystis*, +: Viable *Blastocystis* present, ++: Not counted due to <90% growth inhibition.

**Table 4:** Percentage of growth inhibition of *Blastocystis* in cultures challenged with fractions of *A.* *judaica* (100 µg/mL) against ST1 and ST3 *Blastocystis* subtypes.

Fractions of *A. judaica*	Subtypes of *Blastocystis*			
	ST1		ST3	
EtOAc	100	a	100	a
DCM	100	a	100	a
n-BuOH	92.4	c	95	b
n-hexane	100	a	100	a
Water-soluble fraction *	95	b	94	b

EtOAc: ethyl acetate; DCM: dichloromethane; n-BuOH: n-butanol. Different letters at the same column indicates significant difference (*p* value ≤ 0.5). * Water-soluble fraction was left after partition with different organic solvents.
